# Physiotherapy Rehabilitation for Restoring Function in Quadriparesis After Cervical Spine Trauma: A Case Report

**DOI:** 10.7759/cureus.54073

**Published:** 2024-02-12

**Authors:** Vaishnavi S Sharma, H V Sharath

**Affiliations:** 1 Department of Paediatric Physiotherapy, Ravi Nair Physiotherapy College, Datta Meghe Institute of Higher Education and Research, Wardha, IND

**Keywords:** lower limb paralysis, physiotherapy, rehabilitation, quadriparesis, traumatic cervical spine injury

## Abstract

A major neurological disorder with major socioeconomic effects is spinal cord injury (SCI). This case report aims to document the comprehensive physiotherapy rehabilitation intervention employed in the successful restoration of function in a patient with quadriparesis following cervical spine trauma. An 84-year-old male presented with quadriparesis resulting from a traumatic cervical spine injury sustained in a motor vehicle accident. The patient underwent an individualized physiotherapy rehabilitation program consisting of assessment, goal-setting, and targeted interventions. The rehabilitation plan included a combination of a range of motion exercises, strengthening exercises, neuro-muscular re-education, and functional training. The patient's progress was regularly monitored, and adjustments were made to the rehabilitation program as needed. Over the course of the rehabilitation program, the patient demonstrated significant improvements in muscle strength, joint range of motion, and overall functional abilities. Objective measures, such as manual muscle testing and goniometry, were used to track progress. The patient regained independence in activities of daily living, such as self-care and mobility, and exhibited enhanced motor control and coordination. This case report highlights the efficacy of a tailored physiotherapy rehabilitation approach in restoring function in a patient with quadriparesis following cervical spine trauma. The successful outcomes suggest that a comprehensive and individualized rehabilitation program can significantly contribute to the recovery of individuals with similar conditions. Further research and documentation of such cases may provide valuable insights into optimal rehabilitation strategies for individuals with cervical spine injuries and associated neurological deficits.

## Introduction

Traumatic cervical spine injuries resulting in quadriparesis present significant challenges to both patients and healthcare professionals. These injuries, often caused by accidents or severe trauma, can lead to impaired motor function and diminished quality of life. In such cases, the role of physiotherapy becomes pivotal in guiding individuals through a comprehensive rehabilitation process aimed at restoring functionality and promoting independence. This case report delves into the intricate details of a specific patient who experienced quadriparesis following a traumatic cervical spine injury. Through a multidisciplinary approach, including physiotherapy interventions, this report aims to shed light on the strategies employed to address the unique challenges posed by quadriparesis and document the progressive journey toward functional recovery.

Spinal cord injury (SCI) is a severe neurological disorder that significantly impacts both the affected individuals and the medical system [1]. Over 90% of SCI cases are caused by traumatizing events like violent crimes, sports, falls, or auto accidents, with a 2:1 male-to-female ratio and more common in adults [2]. Women are more vulnerable in their youth (15-19 years old) and seventh decade of life, while men are primarily affected in early and late adulthood (third and eighth decades). The age distribution is bimodal, with young adults comprising the first peak and individuals over 60 making up the second peak. Patients older than 60 have worse outcomes, with falls and aging-related bone abnormalities being common causes [3].

SCI is often caused by a sudden, intense hit to the spine, causing fractures or dislocations of vertebrae. Primary damage occurs when displaced bone fragments, disc materials, or ligaments tear into spinal cord tissue [4]. There are four key harm characteristic processes that have been identified: (1) impact + continuous compression, (2) impact alone with temporary compression, (3) distraction, and (4) tearing or severing [5]. Traumatic cervical spine injuries resulting from deformation of the cervical spinal column can cause spinal cord damage, ranging from minor ligamentous injuries to severe osteo-ligamentous instability with spinal cord compression, causing a wide range of injuries [6]. Traffic accidents, falls, sports-related injuries, and diving mishaps primarily cause cervical spine injuries. Still, non-traumatic injuries can also result from compression fractures due to arthritis, malignancy, or spinal cord inflammation [7]. Many symptoms, including neck pain, restricted range of motion, and neurological abnormalities, can result from injuries to the cervical spine [8-12].

This case report not only highlights the intricate physiotherapy strategies employed but also aims to contribute valuable insights into the broader understanding of effective rehabilitation protocols for individuals facing similar challenges. Through a detailed examination of the patient's journey, we seek to underscore the significance of physiotherapy in fostering recovery, rebuilding strength, and ultimately restoring function in the aftermath of traumatic cervical spine injuries resulting in quadriparesis [13,14].

## Case presentation

Patient information

One week ago, an 84-year-old male patient reported feeling well before experiencing a series of slips and falls in his bedroom, resulting in injuries to his head, neck, and right shoulder. Following this incident, the patient developed sensory loss below the umbilicus and suddenly experienced pain in the right shoulder, accompanied by weakness in both the upper and lower limbs. Consequently, the patient became unable to independently void urine since the day of the injury. Initially, conservative management was pursued, and subsequently, the patient was referred to Acharya Vinoba Bhave Rural Hospital (AVBRH), Sawangi, Wardha, for further investigations. An MRI of the cervical spine revealed significant degenerative changes with spinal cord edema. Subsequently, the patient was referred to the physiotherapy department, where a rehabilitation protocol was initiated.

Clinical finding

The timeline of events is mentioned in (Table 1). The patient was conscious and oriented prior to performing the neurological examination. The consent was obtained from a patient. Vitals were unremarkable. He was assessed in a supine lying position. Upon inspection, it was observed that the patient is supine, lying with the head end elevated to 20 degrees. Patient’s hands and legs were extended and slightly abducted, with a Foley's tube in situ. On the day of evaluation, the Glasgow Coma Scale (GCS) score was 15/15 (E4V5M6). On palpation, the local skin temperature was not raised. The muscle tone is shown in Table 2, manual muscle testing (MMT) results are presented in Table 3, and reflexes are displayed in Table 4. During sensory examination, the patient responds to painful stimuli. The neurological level of injury is at the T8 level.

**Table 1:** Timeline of events AVBRH, Acharya Vinoba Bhave Rural Hospital

Incidents	Dates
Slip and fall	17/12/23
Date of admission in AVBRH	18/12/23
Date of physiotherapy start	22/12/23

**Table 2:** Muscle tone The tone grading system of the upper and lower extremities of the patient; 2+: normal tone.

Muscle tone	Right side	Left side
Upper limb	2+	2+
Lower limb	2+	2+

**Table 3:** Manual muscle testing Manual muscle testing of the upper limb and lower limb of the patient; 3: active movement against gravity. MMT, manual muscle testing

MMT	Right side	Left side
Upper limb	NA	3/5
Lower limb	3/5	3/5

**Table 4:** Reflexes Reflexes of the upper and lower extremities and reflexes of the patient; 0: no reflex, 1+: diminished, 2+: normal.

Reflexes	Right side	Left side
Biceps	NA	+
Triceps	NA	+
Supinator	++	++
Knee	NA	NA
Ankle	0	+
Plantar	0	0

Diagnostic assessment

**Figure 1:**
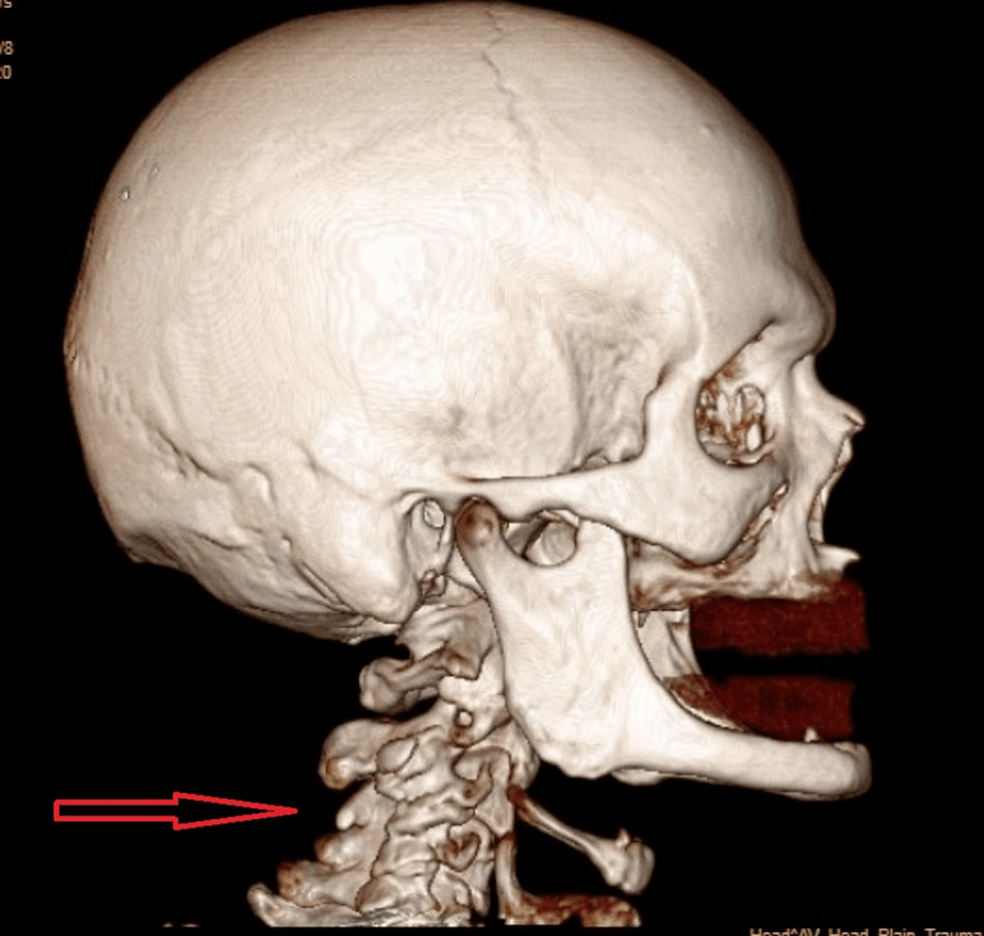
MRI

MRI of the cervical spine reveals significant degenerative changes of the cervical spine with spinal cord edema and significant degenerative changes of the cervical spine. This suggests that there are notable wear and tear, aging-related changes, or structural alterations in the cervical spine. Degenerative changes can include things like disc degeneration, osteophyte (bone spur) formation, and facet joint arthritis. Spinal cord edema indicates swelling or inflammation of the spinal cord. This can be a result of various factors such as compression of the spinal cord, injury, infection, or other inflammatory conditions (Figure 1).

Management

Medical management is given in Table 5, along with its duration. Therapeutic management is given in Table 6, along with the procedure and goals.

**Table 5:** Medications BD, twice a day; OD, once daily; TDS, three times daily

Medication	Dosage	Duration
1. Tab Limcee	500 mg	BD
2. Tab Calcium	500 mg	OD
3. Tab Pan	40 mg	OD
4. Tab Dolo	650 mg	BD
5. Inj Dexa	4 mL IV	TDS
6. Inj Eldervit	2 mL IV	OD

**Table 6:** The goal-oriented physiotherapy protocol

Goals	Therapeutic intervention	Treatment protocol
1. Patient education	In the process of educating the patient about their illness and highlighting the significance of physical rehabilitation, including its potential effects and the preventive measures implemented during the rehabilitation process, this comprehensive approach aims to ensure that both the patient and their family are well-informed and actively engaged in the rehabilitation journey.	Patient education is crucial for understanding the significance of appropriate body alignment, leading to better outcomes and faster recovery.
2. To avoid secondary consequences like deep vein thrombosis and bed sores	To minimize the pressure due to prolonged immobility.	Bed mobility exercise, ankle-toe movement, and changing the position every two hours; used water and air bed to reduce pressure and other complications.
3. To prevent respiratory complication	To improve respiratory function and enhance overall lung capacity.	Techniques used include breathing exercises, pursed lip breathing, thoracic expansion, diaphragmatic breathing, and suctioning.
4. To improve mobility	To minimize the prolonged immobility effect.	Supine, side lying down, side sitting, bed rolling, and bilateral lower limb passive motions; back extension: use as much help as possible.
5. To reduce the tightness, stiffness, and contractures, improve range of motion, and enhance circulation	Stretching for lower limb muscles.	Stretching the hamstrings and tendo achilles (TA) (15-second hold × three reps); passive movements, range of motion.
6. To re-educate muscle	To improve voluntary motor response and motor awareness.	Both upper and lower limb active aided range of motion exercises and static exercises (10 × three reps).
7. To strengthen the pelvic muscles	To regain bowel and bladder control.	Kegel’s exercises, transverse abdominal contraction, hip abductor, and adductor roll (10 × three reps).
8. To control the trunk	To reduce back pain and improve trunk balance and posture.	Exercises for posterior pelvic tilting, hip rotations, and pelvic bridging; exercises for protraction and retraction of the shoulders; static balancing drills that started with sitting and moved up to standing.
9. Sensory integration therapy	To restore sensation, enhance proprioception, enhance functional abilities, reduce the risk of injury, promote psychosocial well-being, and facilitate neuroplasticity.	Using different textures, such as feathers, cotton cloth, Turkish cloth, rubbing sand, silk cloth, etc., from distal to proximal.
10. To strengthen the upper limb and lower limb	It helps to increase activities of daily living (ADL), boost self-esteem, and restore muscular tone.	Low squats, split squats, leg extensions, static lunges, and lateral shoulder raises are examples of static, isometric exercises (10 × three reps).
11. To improve balance	To maintain stability and posture while standing and walking.	Maintaining static balance (20-second hold with eyes closed and open × three reps), shifting weight side to side, forward, and backward while seated (10 reps × one set); putting the feet together while standing.
12. Gait training	To improve walking patterns, promote independence and mobility, and improve joint flexibility.	Frenkel’s exercise in standing; tandem walking.

Follow-up and outcome measures

The follow-up and outcome measures are presented in Table 7.

**Table 7:** Follow-up and outcome measures

Sr. no.	Outcome measures	Month 1	Month 2	Month 3	Month 4
1	Manual muscle testing	2/5	3/5	4/5	5/5
2	Functional independence scale	1/7	3/7	5/7	7/7

## Discussion

Quadriparesis resulting from cervical spine trauma is a complex and debilitating condition that demands comprehensive rehabilitation strategies. The cervical spine, a critical component of the human skeletal structure, plays a pivotal role in facilitating movement and maintaining stability. When traumatic injuries compromise this region, it often leads to neurological deficits, with quadriparesis impacting all four limbs. This case report delves into the intricate physiotherapy strategies employed to restore function in a patient grappling with quadriparesis subsequent to cervical spine trauma. The discussion encompasses the significance of this topic, the extensive scope of physiotherapy interventions, encountered challenges, outcome assessment, the importance of a multidisciplinary approach, patient-centric care, and potential implications for future research. The importance of addressing quadriparesis after cervical spine trauma cannot be overstated. Beyond the immediate physical implications, this condition profoundly affects an individual's autonomy and quality of life. Restoring function in such cases is not merely a therapeutic endeavor but a pursuit of holistic well-being. Successful rehabilitation translates into regained independence and improved daily functioning for individuals grappling with the aftermath of cervical spine trauma-induced quadriparesis [15-19].

Physiotherapy stands at the forefront of rehabilitative efforts for individuals with quadriparesis following cervical spine trauma. The multifaceted approach includes tailored exercises targeting a range of motion, muscle strengthening, coordination training, and balance exercises. Gait training, often a cornerstone of rehabilitation, aims to re-establish a proper walking pattern. These interventions collectively address the diverse challenges presented by quadriparesis, promoting neurological recovery and functional restoration. The rehabilitation journey is fraught with challenges, ranging from the severity of the initial injury to the psychological impact on the patient. Physiotherapists must navigate these challenges with sensitivity and adaptability. Addressing pain management, mitigating fear associated with movement, and effectively managing spasticity are integral components of the rehabilitation process. Patient compliance and motivation also pose ongoing challenges that require a tailored and patient-centered approach [20].

Objective and systematic outcome assessment is indispensable for evaluating the effectiveness of physiotherapy interventions. Functional assessments, muscle strength testing, and monitoring improvements in activities of daily living provide quantitative measures of progress. This meticulous evaluation not only informs the therapeutic approach but also serves as a source of motivation for the patient, reinforcing the importance of their active participation in the rehabilitation process. Recognizing the complexity of quadriparesis after cervical spine trauma, a collaborative and multidisciplinary approach is paramount. Neurologists contribute insights into the neurological recovery process, orthopedic surgeons provide guidance on surgical interventions when necessary, and occupational therapists offer expertise in upper limb rehabilitation. This collaborative effort ensures a comprehensive and well-coordinated approach to address the diverse aspects of quadriparesis rehabilitation.

A patient-centered approach is the cornerstone of successful rehabilitation. Tailoring interventions to the individual's unique needs and goals fosters a sense of agency and active participation. Patient education plays a pivotal role in enhancing understanding and adherence to the rehabilitation program. Empowered patients are more likely to take ownership of their recovery journey, contributing to more favorable outcomes. The landscape of physiotherapy is dynamic, and ongoing research is essential for refining and advancing rehabilitation strategies. Exploring emerging technologies, such as virtual reality or robotics, holds promise for innovative rehabilitation approaches. Additionally, investigating the optimal timing for interventions and refining individualized treatment protocols can further enhance outcomes, paving the way for continuous improvement in the field.

## Conclusions

In conclusion, this case report underscores the crucial role of physiotherapy in restoring function in quadriparesis following cervical spine trauma. By addressing the multifaceted challenges through a comprehensive and patient-centric approach, physiotherapists contribute significantly to the rehabilitation process. The case not only provides valuable insights into the specific strategies employed but also emphasizes the need for ongoing collaboration, research, and innovation in the realm of physiotherapy for complex neurological conditions.
